# Strong Faraday Rotation Based on Localized Surface Plasmon Enhancement of Embedded Metallic Nanoparticles in Glass

**DOI:** 10.1002/smsc.202100094

**Published:** 2022-01-05

**Authors:** Han Zhu, Mingsheng Gao, Chi Pang, Rang Li, Lingrui Chu, Feng Ren, Wei Qin, Feng Chen

**Affiliations:** ^1^ School of Physics State Key Laboratory of Crystal Materials Shandong University Jinan 250100 China; ^2^ Institute of Ion Beam Physics and Materials Research Helmholtz-Zentrum Dresden-Rossendorf 01328 Dresden Germany; ^3^ Department of Physics Center for Ion Beam Application and Center for Electron Microscopy Wuhan University Wuhan 430072 China

**Keywords:** Faraday rotation, integrated photonic devices, ion implantation, localized surface plasmon resonance, orbital angular momentum

## Abstract

The Faraday rotation originates from the mechanism by which the time‐reversal symmetry in the material is broken by the external magnetic field. It is the basis for the development of some magneto‐optical devices, such as optical isolators. In integrated optics and telecommunications, nonreciprocal photonic devices with high transmittance and strong Faraday rotation are desired for low‐cost, compact optical systems. Localized surface plasmon resonance (LSPR) from the metallic nanoparticles in dielectrics allows the light localization in subwavelength scales, boosting the interaction between nanoparticles and materials, which results in a number of plasmon‐enhanced effects. Herein, the strong Faraday rotation in BK7 glass by embedded metallic nanoparticles through LSPR is reported. It is elucidated that the mechanism of Faraday rotation is the near‐field enhancement of spin–photon coupling effect in the presence of an external magnetic field. The Verdet constant of the thin‐layer BK7 glass with embedded Au nanoparticles is determined as high as 5059.7 rad T^−1^ m^−1^ at 532 nm, exhibiting excellent magneto‐optical features. This work opens a new avenue to develop the subwavelength magneto‐optical devices with embedded metallic nanoparticles.

## Graphical abstract

A significant enhancement of Faraday rotation under the condition of local surface plasmon resonance of embedded nanoparticles prepared by ion implantation technology is achieved. It clarifies that the enhancement mechanism is derived from the strong enhancement of the spin–photon coupling effect by the significantly improved near‐field between the particles.

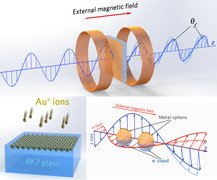

## 1 Introduction

Noble metallic nanoparticles (NPs), e.g., gold (Au) or silver (Ag), exhibit intriguing features due to the localized surface plasmon resonance (LSPR) effect in dielectric environments under light excitation.^[^ ^1^, ^2^, ^3^, ^4^, ^5^ ^]^ The LSPR of light‐excited of noble metal NPs has been widely employed in various applications, including biosensing,^[^ ^6^, ^7^ ^]^ solar cells,^[^ ^8^, ^9^ ^]^ optical imaging,^[^ ^10^, ^11^, ^12^ ^]^ and ultrafast lasers.^[^ ^13^ ^]^ Electron resonance on the surface of NPs leads to significant electromagnetic field amplification in the near‐field region of the frequency band near the resonance frequency, which is the basis of various surface‐enhanced spectroscopy.^[^ ^14^, ^15^, ^16^, ^17^, ^18^ ^]^ Embedded NPs have achieved great success in the tailoring of optical properties in linear and nonlinear processes due to the strong interaction with the dielectric material and the stability that is protected by the surrounding materials.^[^ ^19^, ^20^ ^]^ Ion implantation has been demonstrated as a well‐developed technology to synthesize embedded NPs in dielectrics.^[^ ^21^, ^22^, ^23^, ^24^, ^25^ ^]^ By appropriately selecting the implantation and postannealing parameters, the size, shape, and distribution of NPs can be effectively manipulated, which shows the significant potential for applications in materials design and development of integrated photonic devices.

The Faraday rotation effect with nonreciprocity of light polarization either by reflection or by transmission is the basis of many magneto‐optical devices.^[^ ^26^, ^27^, ^28^, ^29^ ^]^ The interaction between electrons and electromagnetic fields inside nanostructures provides the possibility of combining plasmon and magneto‐optical effects.^[^ ^30^, ^31^, ^32^, ^33^, ^34^ ^]^ In this realm, Belotelov et al. demonstrated that by covering a smooth iron garnet film with nanostructured noble metals, the parameter *δ* that characterizes the transverse magneto‐optical Kerr effect can be enhanced by three orders of magnitude.^[^ ^35^ ^]^ Prashant et al. reported that the optical Faraday rotation in plasmonic colloids is enhanced due to the spectral overlap of the surface plasmon resonance in gold and the electronic transition in maghemite.^[^ ^36^ ^]^ Significant enhancement of the Faraday effect of surface plasmons in nanostructures contributes to the miniaturization and integration of magneto‐optical devices in integrated optical circuits. In addition, some theoretical studies have also predicted the large Verdet constant of noble metal NPs near the LSPR frequency.^[^ ^36^, ^37^ ^]^ Although surface plasmons have shown great potential in the enhancement of magneto‐optical effects, it is still difficult to deeply understand the mechanism behind the phenomena and obtain pronounced Faraday effect with high transmittance. The BK7 borosilicate glass is a widely used material for high‐quality optical components owing to its broadband transparency and relatively low cost.^[^ ^38^, ^39^ ^]^ The development of high‐performance BK7‐based devices plays a critical role in realization of low‐cost compact optical systems. Nevertheless, the extremely small Verdet constant of BK7 glass limits its practical application as magneto‐optical device in integrated photonics technology.

In this work, we experimentally demonstrate the LSPR‐enhanced strong Faraday rotation by embedded Au/AgNPs in BK7 glass (Au/AgNP:BK7). The coupling between local plasmons can further increase the near‐field intensity at the particle voids to contribute to the large Faraday rotation. Particularly, the AuNPs exhibit a larger Faraday rotation than AgNPs due to the larger orbital angular momentum enhanced the coupling between spin and photon. Our results reveal that the mechanism of localized surface plasmon enhancement of the Faraday effect originates from the resonance enhancement of the nondiagonal component of the dielectric constant, which can be used to tailor the properties of subwavelength nonreciprocal magneto‐optical devices.

## 2 Results and Discussion

### 2.1 Theoretical Analysis

**Figure 1:**
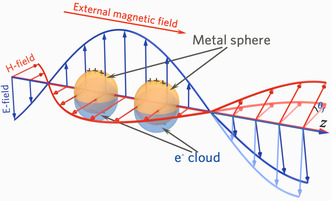
Metallic NPs in the presence of a static magnetic field along the *z*‐axis. LSPR is excited by light propagating along the *z‐*axis.

For spherical NPs embedded in a medium with a scalar permittivity *ε*_s_, when the volume fraction *f* is small, the effective permittivity *ε*_eff_ of the NPs and the medium as a whole can be approximated by the well‐known Maxwell–Garnett formula^[^ ^40^, ^41^ ^]^

(1) $$\frac{\varepsilon_{\text{eff}}-\varepsilon_{s}}{\varepsilon_{\text{eff}}+2\varepsilon_{s}}=f\frac{\varepsilon_{m}-\varepsilon_{s}}{\varepsilon_{m}+2\varepsilon_{s}}$$

where *ε*_m_ is the frequency‐dependent dielectric function of metallic NPs. For a NP placed in the presence of a static magnetic field *B* in the *z*‐axis direction (i.e., the plasmon is excited by light propagating along the *z*‐axis) as shown in **Figure** **1**, its permittivity can be expressed by a complex frequency‐dependent tensor ***ε***_m_^[^ ^42^ ^]^

(2) $$\varepsilon_{m}=(\begin{array}{ccc} \varepsilon (\omega ) & iA(\omega ) & 0\\ -iA(\omega ) & \varepsilon (\omega ) & 0\\ 0 & 0 & \varepsilon (\omega ) \end{array})$$

where the complex frequency‐dependent function *A* = *gB* describes the magnetization‐induced gyration of the material, which originates from the spin–photon coupling effect, and *g* is the Faraday constant. For nonferromagnetic materials, *A* is usually a linear function of magnetic field. Considering NPs with permittivity given by Equation (2), the effective permittivity tensor can be obtained from Equation (1), which can be written as (see Supporting Information for more details)

(3) $$\varepsilon_{\text{eff}}=\varepsilon_{s}I+3f\varepsilon_{s}(1-\gamma^{-1})I+f\gamma^{-2}\alpha_{r}$$

(4) $$\gamma =1-\frac{\Delta \varepsilon }{3\varepsilon_{s}}$$

where ***I*** is the 3 × 3 identity matrix, Δ*ε* = *ε*_s_ − *ε*, and ***α***_r_ = ***ε***_m_ − *ε**I*** is an antisymmetric 3 × 3 matrix that represents the Faraday effect of NPs. Equation (4) shows that, for a medium with scalar permittivity *ε*_s_, *γ* only depends on the diagonal component of the dielectric function of the metallic NPs. The movement of the free electron gas excited by the light field in LSPR changes the electric field distribution in NPs region, resulting in frequency‐dependent dispersion characteristics. Thus, the diagonal component *ε* of the dielectric response of the NP is affected by the collective resonance of the surface electrons bound by the surface of the particle (as shown in Figure 1), which is modulated by the electric field of light. According to Equation (3), the nondiagonal component of the magnetization‐induced gyration in the dielectric response of the medium embedded with NPs can be significantly enhanced as the Fröhlich condition Re[*γ*] ≈ 0 is satisfied.^[^ ^43^ ^]^ This significant enhancement of the Faraday effect is attributed to the significant electric field amplification of the inner and outer near‐field regions of the NPs near the resonance peak of the surface plasmon relative to the incident field.

### 2.2 Sample Characterization

**Figure 2:**
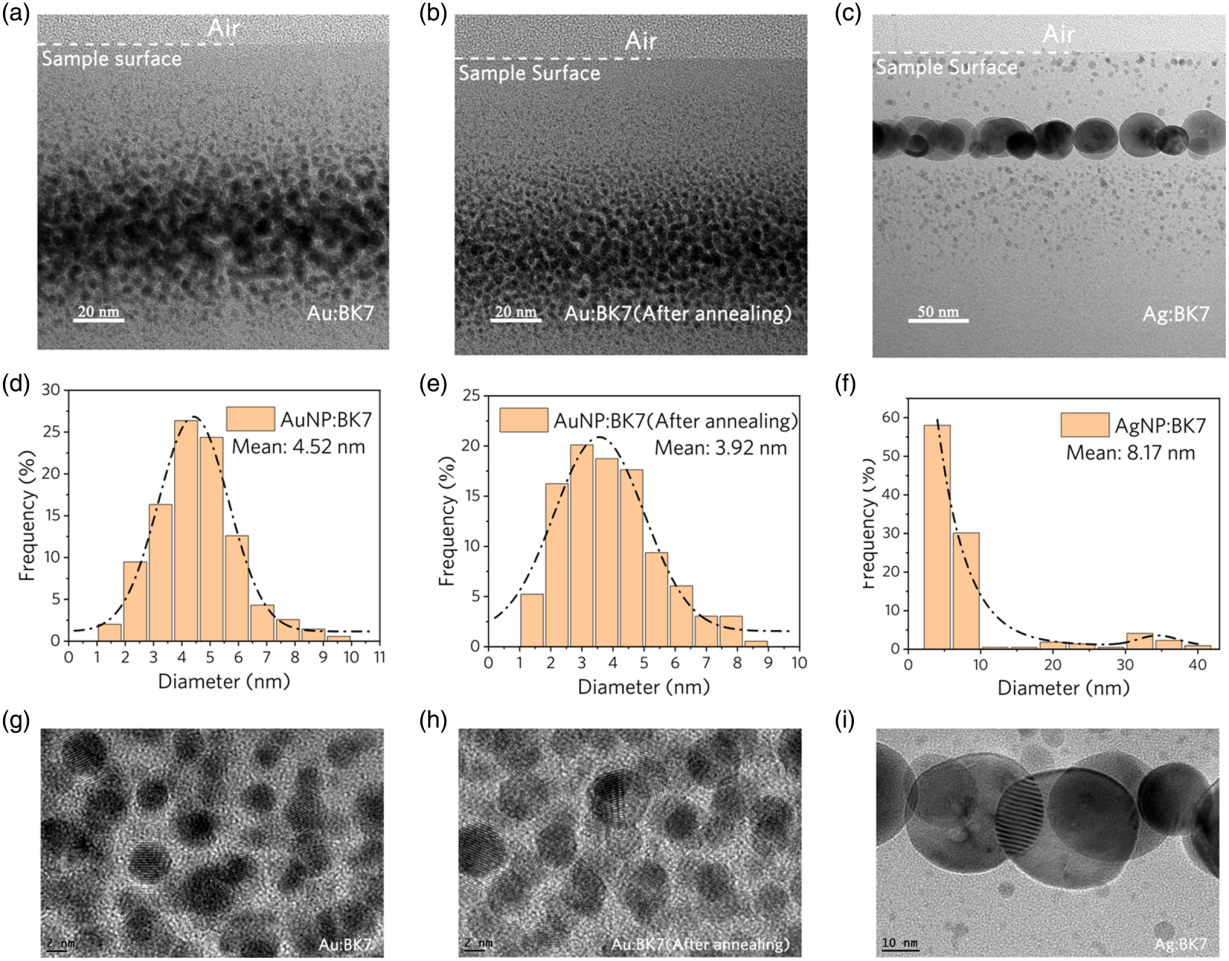
Size analysis and morphological characterization of metallic NPs. a–c) Cross‐sectional TEM micrographs of samples 1–3. d–f) Diameter distribution of NPs embedded in samples based on the TEM micrographs. g–i) HRTEM images of samples 1–3.

**Figure 3:**
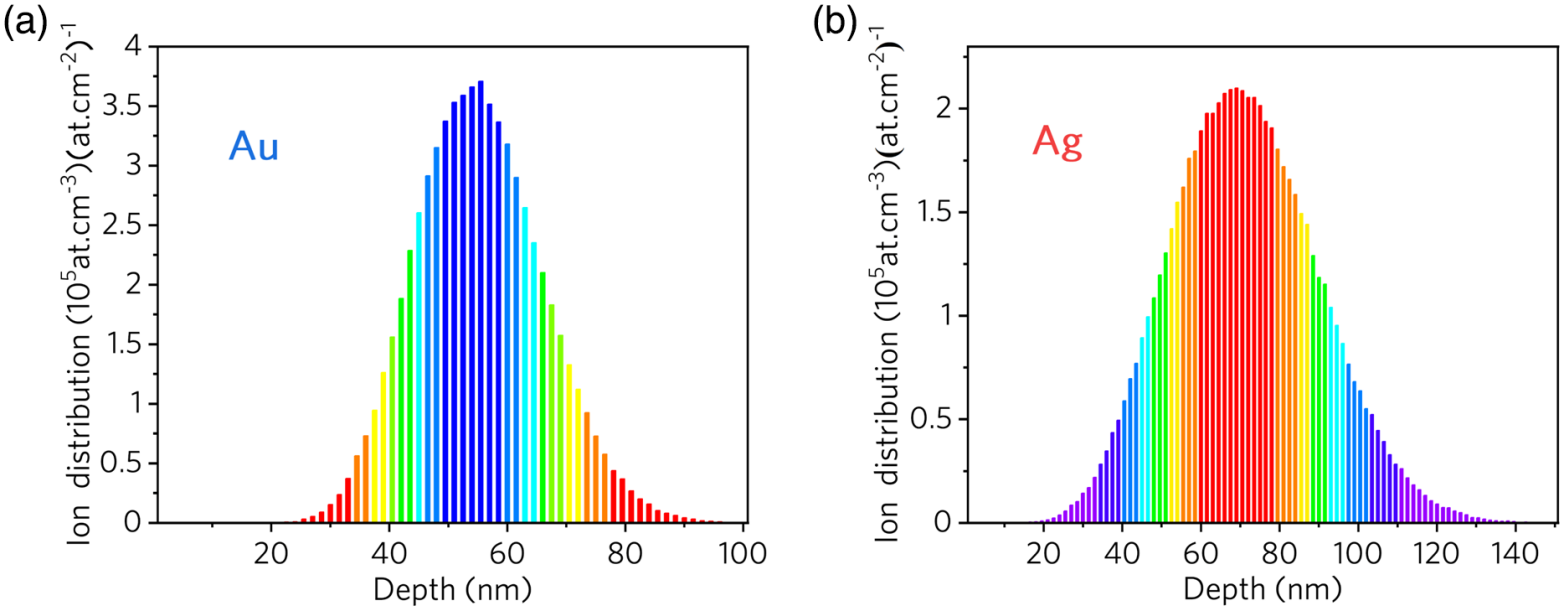
Ion distribution of different elements calculated by SRIM: a) Au and b) Ag.

Based on the strong interaction between embedded NPs and surrounding materials, the above‐described significant enhancement of Faraday rotation due to near‐field amplification can be achieved by fabricated metallic NPs in BK7 glass by direct ion implantation. The detailed sample preparation parameters are shown in **Table** **1** (see Experimental Section for more details). For the observation of the size and morphology of the obtained NPs, transmission electronic microscopy (TEM) micrographs of the cross section of samples are shown in **Figure** **2**a–c. It can be seen that the Au/Ag NPs are located in the region from the sample surface to 100/140 nm depth. The doping of NPs is limited in this 100/140 nm thin layer with high ion concentration, which is in good agreement with the results obtained by the numerical simulation through Stopping and Range of Ions in Matter (SRIM) code in **Figure** **3**a,b.^[^ ^44^ ^]^ The diameter distribution of NPs obtained by statistical analysis of the TEM images is shown in Figure 2d–f. It is obvious that large‐size Ag NPs are formed in Ag:BK7 under high ion fluence. It should be noted that the average size of Au NPs in Au:BK7 decreases after annealing. The explanation for this phenomenon is that the increased temperature during the annealing process reduces the threshold concentration of gold nucleation in BK7, which causes the monomer concentration introduced in the previous implantation process to be supersaturated and aggregates to form smaller NPs, resulting in an average diameter reduction of the NPs in sample 2.^[^ ^45^ ^]^ Further information of NPs, as illustrated in Figure 2i–h, obtained by utilizing high‐resolution transmission electron microscopy (HRTEM) shows that the morphology of the formed NPs conforms to the spherical approximation of Equation (1).

**Table 1:** Parameters of sample preparation

No.	Implanted ions	Fluence [ions cm^−2^]	Energy [keV]	Annealing
1	Au^+^	3 × 10^16^	160	–
2	Au^+^	3 × 10^16^	160	500 °C (1 h)
3	Ag^+^	1 × 10^17^	160	–

**Figure 4:**
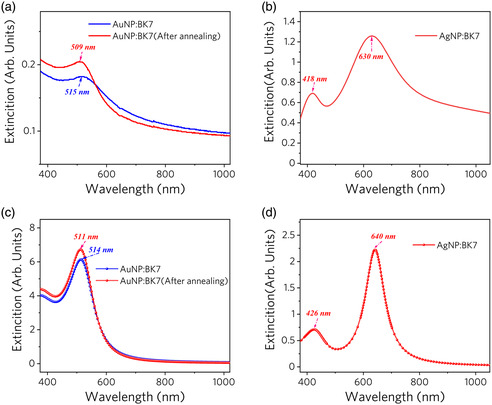
Measurement and simulation of extinction spectra of samples 1–3. Experimental extinction spectrum of the a) Au and b) Ag NPs embedded in BK7. Simulated extinction spectrum of c) Au and d) Ag NPs by COMSOL Multiphysics.

The measured linear optical extinction spectra of samples 1–3 are shown in **Figure** **4**a,b. Different from the LSPR peaks of Au NPs at 509 and 515 nm in samples 1–2, the Ag NPs embedded in sample 3 have two extinction peaks at 418 and 630 nm, which is ascribed to the contribution of nonisolated Ag NPs existing at fluence of 1 × 10^17^ ions cm^−2^ to extinction, as discussed in detail in the study by Li et al.^[^ ^46^ ^]^. As shown in Figure 4a, the LSPR peak of AuNP:BK7 sample shows clear blueshift with intensity enhancement after the annealing, which further demonstrates the formation of small NPs shown in Figure 2b,e. The extinction spectrum is simulated by the finite element method through COMSOL Multiphysics as shown in Figure 4c,d, and the results are consistent with the experimental data.

### 2.3 Faraday Rotation Enhancement

**Figure 5:**
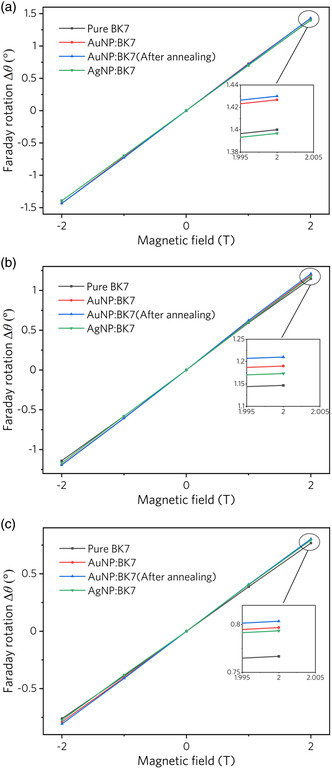
Faraday rotation in samples 1–3 and pure BK7 at wavelength a) 488, b) 532, and c) 635 nm as a function of applied magnetic field.

**Figure 6:**
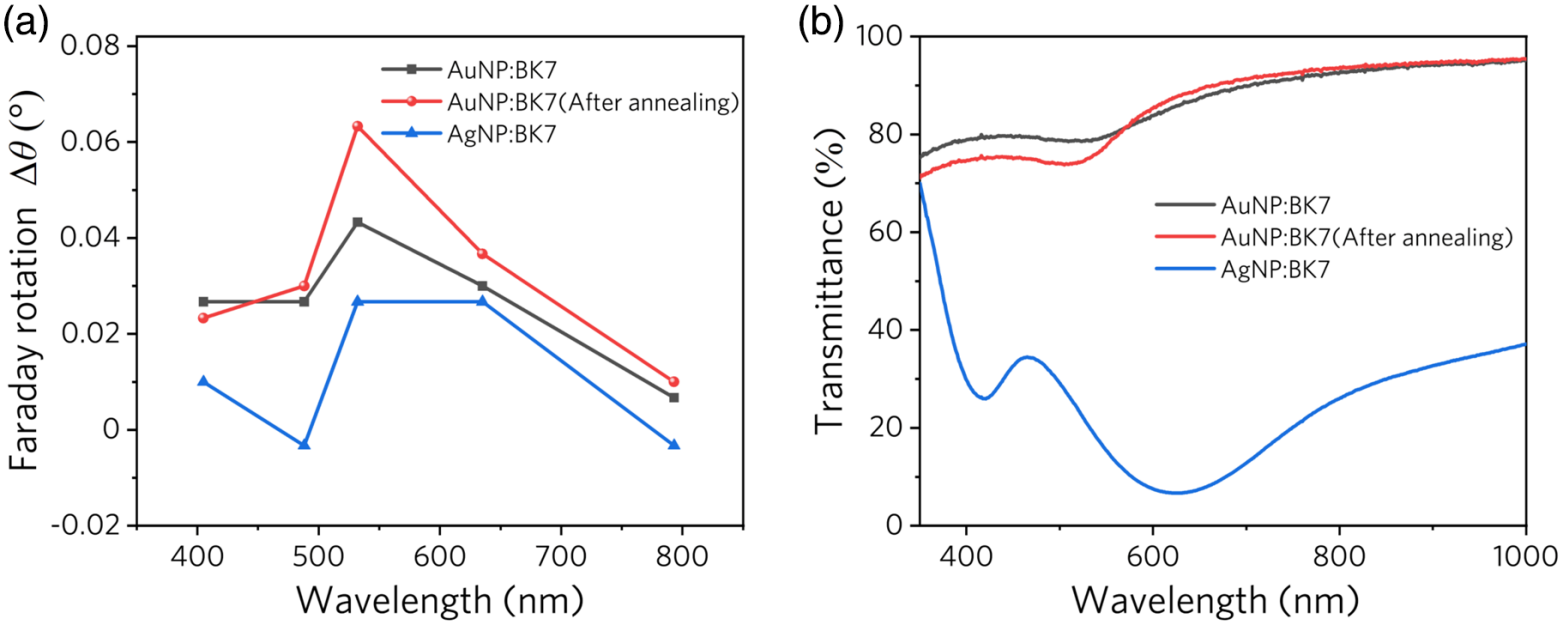
a) Faraday rotation angle affected by BK7 glass has been subtracted. b) Measured transmission spectra of samples 1–3.

**Figure 7:**
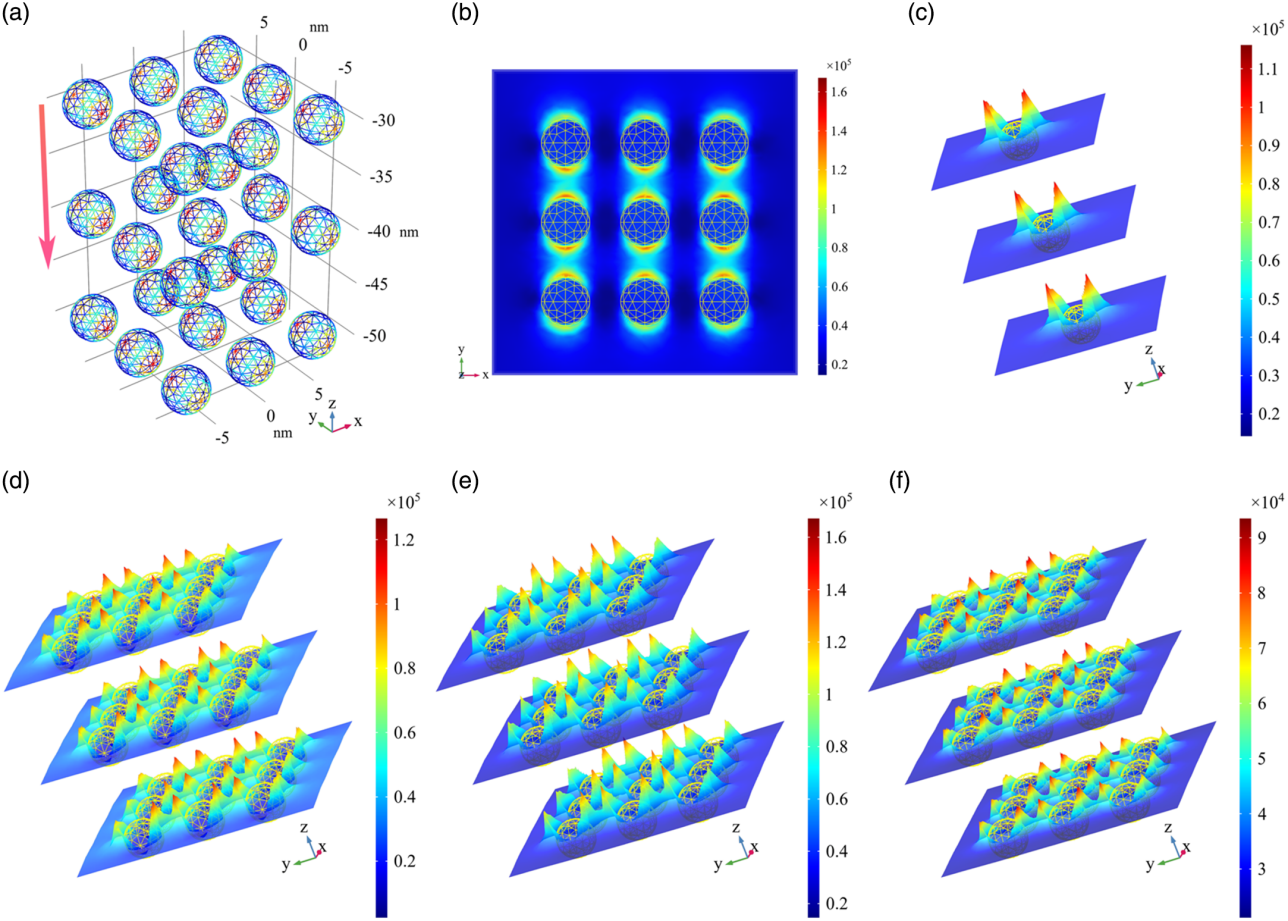
a) The triple‐layer NP model utilized in the simulation, in which localized surface plasmons are excited by light propagating along the *z*‐axis. b,c) Near‐field distribution of single layer and single NP excited by 532 nm wavelength light. The near‐field distribution of NPs under light excitation at d) 488 nm, e) 532 nm, and f) 635 nm.

**Figure** **5**a–c shows the Faraday rotation measured with light and magnetic field perpendicular to the surface of samples 1–3 and pure BK7 at wavelengths of 488 (a), 532 (b), and 635 (c) nm, respectively. As the magnetic field changes from −2 T to 2 T, the Faraday rotation angle increases linearly, and the rotation angle Δ*θ* induced by this applied magnetic field can be described as

(5) $$\Delta \theta =\upsilon B_{\text{app}}l$$

where *B*_app_ is the magnetic induction, *l* is the effective propagation distance of light inside the medium, and *υ* is the Verdet constant. The AuNP:BK7 after annealing at 500 °C for 60 min shows the largest Faraday rotation of 0.8–1.43° at an applied field of 2 T. The rotations of pure BK7, AuNP:BK7, AgNP:BK7 at wavelength of 523 nm in a 2 T magnetic field are 1.15°, 1.19°, and 1.17°, respectively. Compared with Faraday rotation of pure BK7, it is obvious that the Faraday rotation angle of samples with different LSPR extinction spectra has different magnitude increment. As the polarization rotation of the same thickness of pure BK7 has been subtracted from the polarization rotation of the sample, the Faraday rotation angle of the NPs layer under a 2 T magnetic field is obtained as shown in **Figure** **6**a. It can be seen that the Faraday rotation enhancement exhibits resonance feature associated with the extinction of NPs shown in Figure 4. The AuNP:BK7 annealed at 500 °C reaches a maximum Faraday rotation of 0.06° at 532 nm, which is 1308.2 times of magnitude of the Faraday rotation of pure BK7 without NPs (see Supporting Information for details). To further investigate the Faraday rotation resonance enhancement obtained near the LSPR wavelength range of NPs, we construct a triple‐layer NP model based on the practical NPs distribution and simulated the near‐field spatial distribution as shown in **Figure** **7**a. It can be clearly seen that Au NPs generate strong electric field confinement around the surface to contribute to the large Faraday rotation. In addition, the spacing of NPs in our samples is extremely small. The coupling between the localized surface plasmons of embedded NPs makes the scattering between the NPs is strongly suppressed, and the field at the gap is highly localized (see Figure 7b). As shown in Figure 7d–f, the near‐field intensity change trend is consistent with the resonance enhancement of Faraday rotation. It should be noted that compared with the single NP shown in Figure 7c, the coupling effect between NPs corrects the charge distribution of adjacent NPs, resulting in a change in the NPs dielectric response *ε*, thereby significantly improving the near‐field, which means the Faraday rotation induced by NPs in our samples is further enhanced. For the high transparency requirements in potential magneto‐optical applications, we measure the transmission spectra of all samples, as shown in Figure 6b. Obviously, samples 1 and 2 embedded with Au NPs exhibit higher optical transmittance than sample 3 with embedded Ag NPs.

**Figure 8:**
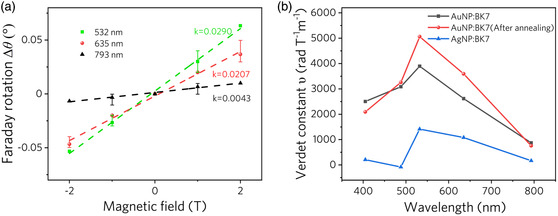
a) Linear fitting of differential rotation angle between sample 2 and pure BK7. b) Verdet constant of the NP layer.

In order to demonstrate the strong Faraday rotation produced by NPs more clearly, we performed a linear fit to the measured differential rotation angle between the samples and the pure BK7 (**Figure** **8**a and Figure S2, Supporting Information), and then the Verdet constant of the NPs layer in samples 1–3 was obtained by Equation (5) (effective distance *l* is the maximum ion range calculated by SRIM shown in Figure 3), as shown in Figure 8b. In this work, the Verdet constant of the Au NPs layer extracted in sample 2 is 5059.7 rad T^−1^ m^−1^ at 532 nm, which is about 51 times that of the previously reported strong Faraday rotation material.^[^ ^47^ ^]^ Based on experimental results shown in Figure 8b, the Verdet constant of Au NPs embedded in thin‐layer BK7 glass is much larger than that of Ag NPs doped sample, which corresponds to the difference in Faraday rotation tensor **α**_r_ of NPs in Equation (3). Besides plasmons resonance of NPs, the orbital angular momentum should be taken into account to further understand the mechanism of Faraday rotation. By using Au NPs instead of Ag NPs, the orbital angular momentum is increased. It is crucial to understand the mechanism of the larger orbital angular momentum dependence of more pronounced Faraday rotation in NPs doped BK7. Generally, the electric field component of light is of apparent interaction with orbital angular momentum, and then affects the spin through the spin–orbit coupling. Once applying external magnetic field, the spin can be polarized in materials, where the polarized spin could indirectly interact with the photon through the channel of spin–orbit–photon with the effect of orbital angular momentum of the NP. As the linearly polarized light can be equivalent to the effect on the combination of the right‐handed *σ*_*+*_ and the left‐handed *σ*_*−*_ circularly polarized light, polarized spins have different interaction intensity with right‐handed and left‐handed component,^[^ ^48^ ^]^ which leads to a difference in propagation speed of the right‐handed and left‐handed circularly polarized light. Therefore, Au NPs with stronger orbit angular momentum could effectively generate larger difference in propagation speed of the right‐handed and the left‐handed circularly polarized light, which results in a more pronounced Faraday rotation.

## 3 Conclusion

In summary, we have observed strong Faraday resonance rotation in Au/Ag NPs embedded in BK7 glass. The small metallic NPs formed during the annealing process increase the LSPR of the sample and produce a blueshift of the peak. The near‐field distribution shows that the coupling of local plasmons in our sample makes the field at the gap highly localized, thereby greatly enhancing the field strength to contribute to the large Faraday rotation. In addition, comparing to Ag NPs, Au NPs with larger orbital angular momentum can enhance the indirect interaction between spin and photon through spin–orbit–photon to lead to a more pronounced Faraday rotation effect. Owing to the matrix flexibility of ion beam technology in the synthesis of NPs, we expect the large Faraday rotation effect by LSPR paves the way for subwavelength nonreciprocal magneto‐optical devices. In further work, our concept could be applied to strong magneto‐optical effect materials, and the integration for devices could be achieved by thinning the samples to the micrometric or even subwavelength scale to reduce the influence from the substrate.

## 4 Experimental Section

4.1

4.1.1

##### NPs Synthesis

Four BK7 glass wafers with a size of 10 × 10 × 2 mm^3^ were optically polished. The Au^+^/Ag^+^ ions at an energy of 160 keV were implanted into the BK7 substrate by ion‐implanter LC22‐1C0‐01 at Wuhan University. The ion fluences for each sample are shown in Table 1. The noble metal ions were randomly embedded in BK7 glass and aggregated to synthesize NPs when the atomic concentration exceeded the nucleation threshold concentration. After implantation, the sample 2 was annealed at 500 °C for 1 h in air.

##### Metallic NPs Characterization

TEM measurements were performed by FEI Tecnai G2 F20 S‐TWIN, with an acceleration voltage of 200 kV and a pixel resolution of 0.24 nm. The linear extinction spectra of the samples were measured by using a UV–vis–NIR spectrophotometer (Hitachi, U‐4100) at room temperature in wavelengths ranging from 350 to 1100 nm. The LSPR absorption spectrum of NPs was obtained by subtracting the absorption of pure BK7 from the absorption of samples 1–3, respectively.

##### Faraday Rotation Measurement

The arrangement of the Faraday rotation measuring apparatus is shown in Figure S2, Supporting information. The sample was loaded in the magnetic field generated by the electromagnet. The laser beam was perpendicular to the sample surface after passing through the linear polarizer. Applied magnetic field can be parallel or antiparallel to the direction of incident light. After the beam passed through the sample, the transmitted light data were collected by PAX1000VIS and PAX1000IR1, Thorlabs. Faraday rotation was deduced from the angle of the rotating analyzer.

##### Numerical Simulation

Numerical simulations of the extinction spectrum and near‐field distribution of NPs were performed using the commercial software COMSOL Multiphysics. The electromagnetic waves were incident perpendicularly from the air to the BK7 glass embedded with NPs. The refractive index of Au NPs was defined by the Lorentz–Drude model. Port boundary conditions and periodic boundary conditions were applied to the boundary perpendicular to the direction of light propagation and other boundaries, respectively.

## Conflict of Interest

The authors declare no conflict of interest.

## Supporting information

Supplementary Material

## Data Availability

The data that support the findings of this study are available from the corresponding author upon reasonable request.
