# Supplementary material for: Strong Faraday Rotation Based on Localized Surface Plasmon Enhancement of Embedded Metallic Nanoparticles in Glass
Source: Small Sci. 2022 Jan 5;2(4):2100094. doi: 10.1002/smsc.202100094 (PMC11935988; doi:10.1002/smsc.202100094)
Supplement: Supplementary file 1 — Supplementary Material [file SMSC-2-2100094-s001.pdf]

Supporting Information

Strong Faraday rotation based on localized surface plasmon enhancement of embedded metallic nanoparticles in glass

Han Zhu, Mingsheng Gao, Chi Pang, Rang Li, Lingrui Chu, Feng Ren, Wei Qin, and Feng Chen**

For the small volume fraction of NPs, Equation 1 can be expanded and retained to the first order of *f*, the result is:

$$\varepsilon_{eff} = \varepsilon_s + 3f\varepsilon_s \frac{\varepsilon_m - \varepsilon_s}{\varepsilon_m + 2\varepsilon_s} \tag{S1}$$

Considering NPs with permittivity given by Equation 2, the effective permittivity tensor can be obtained from Equation S1, which can be written as:[1]

$$\boldsymbol{\varepsilon}_{eff} = \begin{pmatrix} \varepsilon_{11} & iA_{eff} & 0 \\ -iA_{eff} & \varepsilon_{22} & 0 \\ 0 & 0 & \varepsilon_{33} \end{pmatrix} \tag{S2}$$

where

$$\varepsilon_{11} = \varepsilon_{22} = \varepsilon_s + 3f\varepsilon_s \frac{(\varepsilon - \varepsilon_s)(\varepsilon + 2\varepsilon_s) - A^2}{(\varepsilon + 2\varepsilon_s)^2 - A^2} \tag{S3}$$

$$A_{eff} = \frac{9f\varepsilon_s^2 A}{(\varepsilon + 2\varepsilon_s)^2 - A^2} \tag{S4}$$

$$\varepsilon_{33} = \varepsilon_s + 3f\varepsilon_s \frac{\varepsilon - \varepsilon_s}{\varepsilon + 2\varepsilon_s} \tag{S5}$$

Normally, the function *A* is a small amount relative to the diagonal elements ε in Equation 2. A_{eff} can be expanded to the first order of *A*, and Equation S2 can be reduced to a more general form:

$$\boldsymbol{\varepsilon}_{eff} = \varepsilon_s \boldsymbol{I} + 3f\varepsilon_s \frac{\varepsilon - \varepsilon_s}{\varepsilon + 2\varepsilon_s} \boldsymbol{I} + \boldsymbol{\alpha}_r \gamma^{-2} \tag{S6}$$

The element mappings of implanted region of samples 1-3 after ion implantation are shown in **Figure S1**a-c. It can be clearly seen that embedded NPs are formed in the BK7 glass. The selected area electron diffraction (SAED) images of implanted region of samples 1-3 after ion implantation are shown in Figure S1d-f, respectively, which further confirms the formation of NPs in BK7 glass.

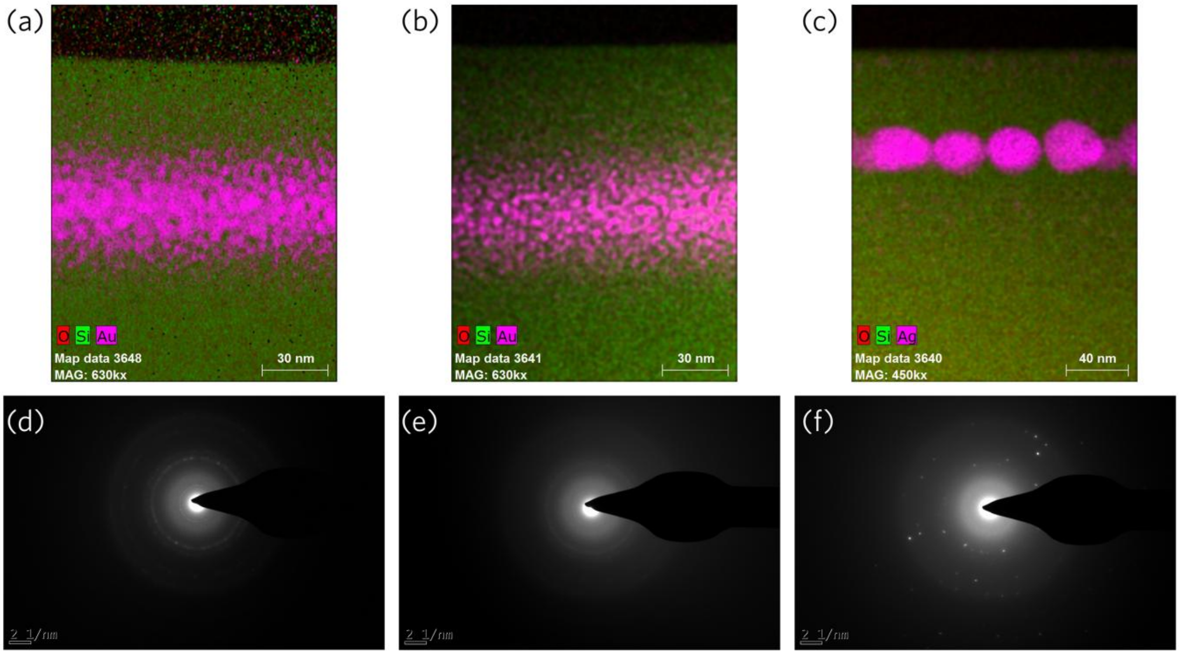

Figure S1. The element mapping and SAED images of samples 1-3 after ion implantation. a,d) Sample 1. b,e) Sample 2. c,f) Sample 3.

Figure S2 shows the Faraday rotation measurement system, the applied magnetic field is parallel or anti-parallel to the direction of the incident light. The laser beam is linearly polarized by the polarizer and then incident perpendicular to the surface (the implanted surface of the samples 1-3) of the sample placed in the electromagnet. The light beam transmitted through the sample is collected by the detector after passing through the attenuator.

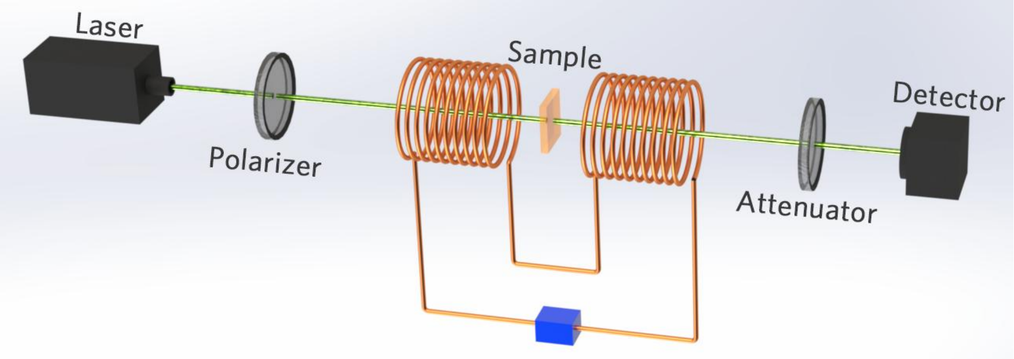

Figure S2. Experimental setup of Faraday rotation measurement system.

The linear fitting of the differential Faraday rotation angle between samples 1-3 and pure BK7 at different wavelengths is shown in **Figure S3**.

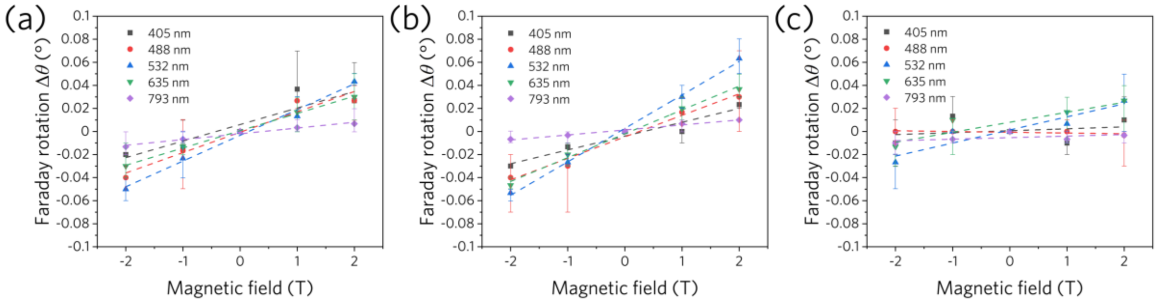

Figure S3. Linear fitting of differential rotation angle between samples 1-3 and pure BK7.

Figure S4 shows the thickness of the samples and pure BK7 glass in this work. It can be seen that the thickness difference between ion implanted sample and pure BK7 glass is less than ~0.01/2.37 = 0.42%.

In a 2T magnetic field at 523 nm, the Faraday rotation angle of pure BK7 glass with a thickness of 2.378 mm is 1.147°, the Faraday rotation angle of the 100 nm Au ion implantation layer is 0.063°, and the enhancement factor is $(0.063/10^{-4})/(1.147/2.378) \approx 1308.2$.

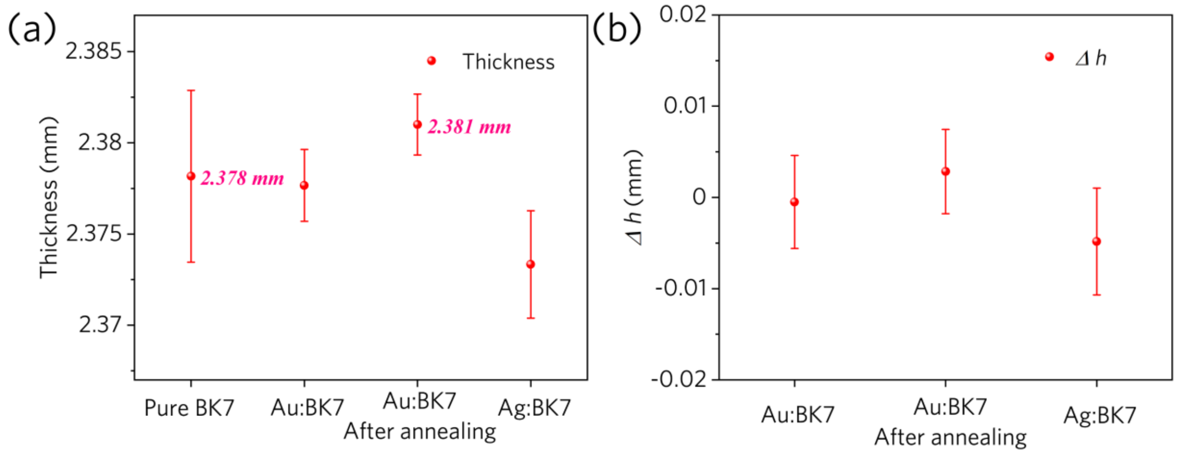

Figure S4. a) The thickness of samples and pure BK7 glass. b) The thickness difference Δ*h* between the samples and pure BK7 glass

Figure S5 shows the Figure of merit (FOM) of ion implantation region (NPs layer) in our samples under 2T magnetic field.[2]

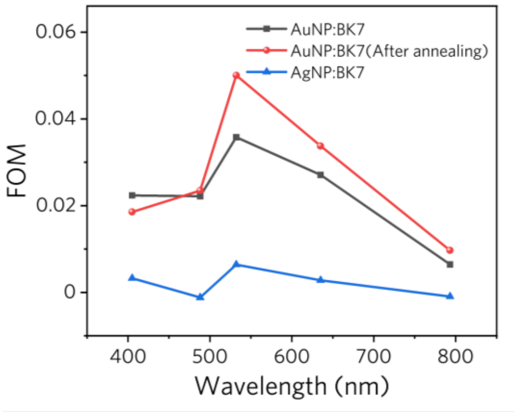

Figure S5. FOM of NPs layer under 2T magnetic field

References

[1] P. M. Hui, D. Stroud, *Appl. Phys. Lett.* **1987**, *50*, 950.

[2] B. Caballero, A. Garcia-Martin, J. C. Cuevas, *Opt. Express* **2015**, *23*, 22238
